# Long-Term Effect against Methicillin-Resistant *Staphylococcus aureus* of Emodin Released from Coaxial Electrospinning Nanofiber Membranes with a Biphasic Profile

**DOI:** 10.3390/biom10030362

**Published:** 2020-02-27

**Authors:** Peiwen Ye, Suying Wei, Chaohua Luo, Qirui Wang, Anzhang Li, Fenghuan Wei

**Affiliations:** 1 College of Traditional Chinese Medicine, Southern Medical University, Guangzhou 510515, China; ypw0108@i.smu.edu.cn (P.Y.); lchua2008@smu.edu.cn (C.L.); wqrui@smu.edu.cn (Q.W.); 2 Department of Chemistry and Biochemistry, Lamar University, Beaumont, TX 77710, USA; suying.wei@lamar.edu; 3 Guangdong Microbial Culture Collection Center, Guangdong Institute of Microbiology, Guangzhou 510515, China; liaz@gdim.cn

**Keywords:** coaxial electrospinning, emodin, nanofiber, MRSA

## Abstract

Methicillin-resistant *Staphylococcus aureus* (MRSA) is a serious and rapidly growing threat to human beings. Emodin has a potent activity against MRSA; however, its usage is limited due to high hydrophobicity and low oral bioavailability. Thus, the coaxial electrospinning nanofibers encapsulating emodin in the core of hydrophilic poly (vinylpyrrolidone), with a hygroscopic cellulose acetate sheath, have been fabricated to provide long-term effect against MRSA. Scanning electron microscopy and transmission electron microscopy confirmed the nanofibers had a linear morphology with nanometer in diameter, smooth surface, and core-shell structure. Attenuated total reflection-Fourier transform infrared spectra, X-ray diffraction patterns, and differential scanning calorimetric analyses verified emodin existed in amorphous form in the nanofibers. The nanofibers have 99.38 ± 1.00% entrapment efficiency of emodin and 167.8 ± 0.20% swelling ratio. Emodin released from nanofibers showed a biphasic drug release profile with an initial rapid release followed by a slower sustained release. CCK-8 assays confirmed the nontoxic nature of the emodin-loaded nanofibers to HaCaT cells. The anti-MRSA activity of the nanofibers can persist up to 9 days in AATCC147 and soft-agar overlay assays. These findings suggest that the emodin-loaded electrospun nanofibers with core-shell structure could be used as topical drug delivery system for wound infected by MRSA.

## 1. Introduction

*Staphylococcus aureus* (*S. aureus*) is one kind of pathogenic microorganism threating human health. With the extensive usage of broad-spectrum antibiotics, *S. aureus* has developed resistance to methicillin since the 1960s [1,2]. Especially, methicillin-resistant *Staphylococcus aureus* (MRSA) has been emerging as one of the most serious threats for hospitalized patients and especially patients in intensive care units [1,3,4], which can not only potentially lead to a lethal sequence of burn wound sepsis, invasive infection, and death [5] but also colonize in some burn patients causing additional complications [6]. Moreover, vancomycin, once considered as the last defense line, also fails to control some *S. aureus* strains [7]. The emergence and worldwide spread of MRSA represent some of the most important events in the epidemiology of infectious diseases [1]. Therefore, it is urgent to develop novel drug delivery systems against MRSA.

**Figure 1:**
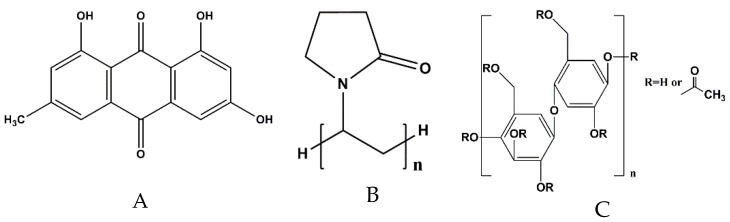
Structures of emodin and other excipients. (**A**) Emodin, (**B**) poly(vinyl pyrrolidone), (**C**) cellulose acetate.

Emodin,1,3,8-trihydroxy-6-methyl-anthraquinone (Figure 1A), a common chemical component found in many natural plants [8], has outstanding antimicrobial [9,10], anticancer [11], antioxidant [12], and anti-inflammatory [13,14] activities. It has attracted enormous attention due to its potent inhibition effect on methicillin-sensitive *S. aureus* and MRSA related to pyruvate pathway imbalance induction, protein synthesis inhibition, and DNA synthesis suppression [10]. However, its usage is limited due to its poor solubility in water and low oral bioavailability because of extensive glucuronidation in liver and intestine [12,15,16]. To improve its water solubility and bioavailability, various emodin-loaded delivery systems have been reported, such as nanoparticle [17], thermo-reversible gel [18], nanoemulsion [19], microspheres [20,21,22,23], liposome, layer-by-layer film [24], and electrospinning fiber membrane [25]. Among those delivery systems, electrospinning nanofiber membranes are a promising choice for wound dressing. However, most reported nanofiber membranes fabricated by monoaxial electrospinning mode showed an extreme burst release profile, for example, the cumulative release was 96.7% during the first 60 min; after 90 min, emodin was completely released, so fresh nanofibrous membranes had to be reapplied every other day on test animals for wound healing [25]. Therefore, it is necessary to optimize electrospinning conditions for the development of nanofiber membrane emodin delivery system with sustained release profiles.

Electrospinning is a technique processing solutions or melts (mainly of polymers) into continuous fibers with diameters ranging from a few micrometers to a few nanometers [26], and the electrospinning nanofibers have unique characteristics compared to other nano-structures: (a) high porosity similar to the natural extracellular matrix (ECM) is favorable for cell adhesion, proliferation, and migration [27,28]; (b) large specific surface is favorable for wound exudate, drug dispersion, and enhancing solubility of poorly water-soluble drugs [26,29]; (c) fiber morphology is favorably used as multifunctional material for wound dressing applications. Especially, by controlling solution properties (mainly including types of polymers, solvents and concentration of polymers), electrospinning modes (mainly including blending electrospinning, coaxial electrospinning, and sequential electrospinning), and electrospinning parameters (mainly including voltage, flow rates, and receiving distance), we can modulate: (a) fiber composition, (b) fiber diameter and micro- or nanometer dimensions, (c) fiber morphology (e.g., smooth, wrinkled, porous, etc.), (d) fiber structure (e.g., blended fibers, shell-core fibers, layer-layer fibers, etc.), and (e) drug release profiles. Among the above-mentioned electrospinning conditions, electrospinning mode is one of the key factors in controlling drug release from electrospinning nanofibers, because blending electrospinning can only yield single homogenous nanofibers containing drug and polymers which easily results in the burst release of the drugs from the fibers, while coaxial electrospinning could produce fiber with a core–shell structure, and drug encapsulated in core layer of the fibers can present a sustained release profile on the basis of the thickness and composition of the shell layers [28,30].

In fabrication of drug-loaded delivery systems using electrospinning, the selection of polymers is very important, as biodegradability, biocompatibility, water solubility, and nontoxicity of polymers would determine the properties of the resultant electrospinning fibers. Poly (vinyl pyrrolidone) (PVP, Figure 1B) is an important hydrophilic polymer commonly used in medical or pharmaceutical industries to increase the water solubility of drugs and to inhibit the recrystallization of crystalline drugs. The U.S. Food and Drug Administration has approved PVP for a wide variety of applications and it is generally considered safe. Meanwhile, because of its well spinnable and mechanical properties, PVP can be used alone or combined with other polymers to fabricate electrospinning fibers containing drugs, such as mangostin-loaded PVP nanofibers [31] and naproxen-loaded PVP-ethyl cellulose nanofibers [32]. Cellulose acetate (CA, Figure 1C), an acetate ester derivative of cellulose, possesses advantageous properties in biodegradability, biocompatibility, insolubility in water, and nontoxicity [33], and is also widely used to fabricate drug-loaded electrospinning fibers, such as ibuprofen-loaded nanofibers [34], and nisin-loaded nanofibers [35]. In this paper, PVP and CA were selected as core-layer polymer and shell-layer polymer, respectively.

Antimicrobial materials whose effects can last one week or more will be highly beneficial in many applications [35]. Here, the fabrication of coaxial electrospun nanofibers encapsulating emodin in the core of PVP with a CA sheath was studied in order to investigate its effect against MRSA over time.

## 2. Materials and Methods

### 2.1. Materials

Emodin (purity, 98.6%) was purchased from Wei Ke Qi Biological Technology Co., Ltd. (Chengdu, China). Poly(vinyl pyrrolidone) (PVP, Mav = 1,300,000) and cellulose acetate (CA, acetyl content of 39.8%; MW = 30 kDa) were purchased from Dalian Meilun Biological Technology Co., Ltd. N,N-Dimethylacetamide (DMAC) and acetone were purchased from Shanghai Macklin Biochemical Co., Ltd. (Shanghai, China). Mannitol salt agar and Methicillin-resistant *Staphylococcus aureus* (MRSA) GDMCC 1.1263 were provided by Guangdong culture collection center (GDMCC 1.1263 = ATCC 43300). HaCaT cells were bought from China Center for Type Culture Collection (CCTCC, Wuhan, China).

### 2.2. Sample Preparation

Two different polymeric solutions were prepared for core and sheath layers. For the core solution, PVP was firstly dissolved in the solvent mixture of acetone and DMAC (2/1, *v*/*v*) using ultrasonic treatment to get 13% PVP solution (*w*/*v*), then emodin was added to the PVP solution and mixed by vortex to obtain a homogeneous emodin/PVP solution (8.5% emodin relative to PVP, *w*/*w*). For sheath solution, CA was dissolved in the same solvent mixture of acetone and DMAC (2/1, *v*/*v*) using ultrasonic treatment to obtain the homogeneous sheath solution containing 3% CA (*w*/*v*). For the control fiber membrane without emodin, the same ratio of PVP and CA solution was prepared using the above-mentioned method.

The electrospinning equipment (Beijing Ucalery Co., Beijing, China) used mainly consisted of a high-voltage power supply (0–30 kV), two syringe pumps with a coaxial metallic needle of 14G (the inner and outer diameters were 1.55 mm and 2.10 mm, respectively), and a drum collector. Before electrospinning, the core solution and shell solution were transferred into a 2.5 mL syringe, respectively. During the electrospinning process, 11.0–12.5 kV of the voltage was applied, the flow rate of the core solution and the shell solution was both 0.3 mm/min, the distance between the nozzle and the collector was set to 15 cm, and a square aluminum foil (30 × 30 cm) was used as the receiving carrier to be placed on the collector and rotated at the speed of 20 rpm. All electrospinning experiments were carried out at room temperature with a relative humidity of 48%–67%, and the nanofiber membranes were dried in a glass desiccator with silica-gel desiccant at room temperature to dry over 72 h before use.

### 2.3. Morphology of Electrospun Nanofibers

The diameter and morphology of electrospinning fibers with and without emodin were studied by scanning electron microscope (6330F, Tokyo, Japan). Before imaging, the nanofiber membranes were coated with 5 nm Au/Pd by sputtering machine (Cressington Auto 108 Sputter Coater, Ted Pella Inc., Redding, CA, USA), and then images were taken at 1000 to 10,000 magnification. The diameters of the fibers were measured by Image J (ver.1.8.0) software, and a histogram showing the diameter distribution was generated using Origin (ver.8.0) software. The diameters of the nanofibers (n = 100 fibers) were reported as mean ± SD.

Transmission electron microscope (TEM, FEI Tecnai G_2_ Spirit, Eindhoven, The Netherlands) was used to present the core-shell structure of fibers containing emodin. The samples were prepared by a 200 × 200 Cu Mesh, and the TEM was operated at an accelerating voltage of 120 kV using bright field mode.

### 2.4. Structural Characterization of Electrospinning Nanofibers

#### 2.4.1. Attenuated Total Reflection–Fourier Transform Infrared Spectra Analysis

The characteristic functional groups of electrospinning nanofibers containing emodin and the raw materials were detected by a Fourier transform infrared (FTIR) spectra instrument with attenuated total reflection (ATR) (Bruker Tensor II, Karlsruhe, Germany) in the range of 400–4000 cm^−1^ with a resolution of 4 cm^−1^. ATR was a nondestructive analysis technique, which can detect functional groups without destroying the structures of materials.

#### 2.4.2. X-ray Diffraction

To evaluate the existing form of emodin in the core layer, nanofiber membrane was examined with X-ray diffraction (XRD, Empyrean, Panalytical, Almelo, The Netherlands), and to better understand the structural characterization of fiber membrane with emodin, blank fiber membrane and the raw materials were also detected by XRD. The diffracted intensity of Cu Kα radiation was measured in the 3–60° 2θ range with a scan rate of 10°/min.

#### 2.4.3. Differential Scanning Calorimetry

Differential scanning calorimetry (DSC, DSC21400A-0211-L, Netzsch, Selb, Germany) was also used to determine the existence form of emodin in the nanofibers. The nanofiber membranes with and without emodin, and raw materials were tested. The samples were heated at 10 K/min from 20 to 300 °C under nitrogen flow.

### 2.5. Entrapment Efficiency

The entrapment efficiency (*EE*%) of emodin was determined following this procedure: The nanofiber membranes with emodin were weighed accurately in triplicate and extracted in methanol using a rotating stirrer at 20 rpm, and the extract solution was centrifuged at 10,000 rpm for 10 min at room temperature; then, the supernatant was transferred to a cuvette and measured by UV/vis spectrophotometer (UV-Vis 8454, Agilent, Santa Clara, CA, USA) at 254 nm. The methanol solution of raw materials without emodin was used as the control sample solution to avoid background interference. The emodin in the nanofiber membrane was calculated using the calibration curve of emodin determined in the same conditions. *EE* % was calculated using the following equation:

(1) $$EE\left(\%\right)=\frac{W_{e}}{W_{a}}\times 100\%$$

In this equation, *EE* is the entrapment efficiency, *W_e_* is emodin determined in nanofiber membranes and *W_a_* represents the emodin added in electrospinning fibers. All measurements were performed in triplicate.

### 2.6. In Vitro Release Profiles

The release profiles of emodin from nanofibers and raw emodin were investigated in PBS at pH 7.4. The fiber membranes (7.61 mg, 7.74 mg, and 7.46 mg, respectively) and raw emodin (0.75 mg, 0.81 mg, and 0.81 mg, respectively) weighed were incubated in 50 mL PBS solution at 37 ± 1 °C. Samples (2 mL) were drawn from the release medium at intervals of 0.17, 0.5, 1, 2, 4, 6, 8, 10, 12, and then every 12 h until the release was complete; subsequently the fresh PBS of the same volume was replaced. The samples were centrifuged at 10,000 rpm for 10 min at room temperature; then, the supernatant was transferred to a cuvette and measured by UV/vis spectrophotometer at 254 nm, and the PBS solution of raw materials except emodin was used as the control sample. The emodin in the liquid drawn at different intervals was calculated using the calibration curve established with standard emodin solutions. All measurements were performed in triplicate.

### 2.7. Swelling Ratio

The swelling ratio is an important parameter for evaluating wound dressings [36] and usually tested using the gravimetric method [37]. In this paper, the nanofiber membranes were cut into 3 × 3 cm and immersed in PBS (pH 7.4, 37 ± 1 °C). At certain intervals, the membranes were taken out and put on a piece of filter paper to remove water adhered to the surface of the membranes before being weighted. The results were reported as swelling ratio (*SR*%) to timeline charts. All measurements were performed in triplicate.

(2) $$SR\left(\%\right)=\frac{W_{w}-W_{d}}{W_{d}}\times 100\%$$

In this equation, *SR* is the swelling ratio, *W_w_* is the weight of sample after swelling, and *W_d_* represents the dry weight of the membrane.

### 2.8. Cell Proliferation Assay

HaCaT cells (Human keratinocyte HaCaT cells line) were used to provide an assessment of cell viability on the emodin encapsulated nanofibers. HaCaT cells were routinely maintained in DMEM medium (Gibco, Carlsbad, CA, USA) supplemented with 10% FBS (Gibco) and antibiotics (100 U/mL of penicillin and 100 μg/mL streptomycin) at 37 °C in a humidified atmosphere containing 5% CO_2_. The cell viability was examined by CCK-8 assays according to the manufacturer’s instructions. Cells were seeded at a density of 2 × 10^3^ per well in 96-well plates (Corning, New York, NY, USA) and cultured overnight. Then, the nanofiber membranes (cut into 1 × 1 mm and sterilized by UV for 30 min before use) were transferred into the 96-well plates with cells and inoculated together. The medium was then replaced with fresh medium containing the membranes. The morphologies of cells were viewed under Eclipse Ti-s microscope (Nikon, Tokyo, Japan). Then, 10 μL of CCK-8 reagent (Dojindo, Kumamoto, Japan) was added to each well and incubated at 37 °C for 1–2 h at each day. The absorbance was measured at a wavelength of 450 nm using a microplate reader ((Multiskan GO, Thermo FC, Waltham, MA, USA) to reflect cell viability; each assay was repeated in triplicate.

### 2.9. Antimicrobial Activity against MRSA

Both AATCC test method 147 and soft agar overlay technique were used to qualitatively assess the activity of emodin-loaded nanofiber membrane, and the control nanofiber membrane was also analyzed in the same way. The nanofiber membranes were cut into small pieces of 2 × 2 cm and 2 × 3 cm, respectively, and disinfected by ultraviolet irradiation for 30 min before use. Methicillin-resistant *Staphylococcus aureus* (MRSA, ATCC 43300) was incubated in nutrient broth on a shaker at 37 °C for 6 h (approximately 10^7^ CFU/mL). For AATCC test method 147, the culture broth was streaked on mannitol salt agar (MSA) plate in six parallel lines spaced about 1 cm apart away from the nearest neighbor. For soft agar overlay assay, 10, 20, and 30 μL of the MRSA culture broth was evenly spread on soft MSA plates, respectively, and allowed to dry. Subsequently, in the same plate, one piece of emodin-loaded membrane and one piece of control membrane without emodin of the same size were placed in the upper side and underside of the plate over the lines and incubated at 37 °C and observed in the following 9 days.

### 2.10. Statistical Analysis

The statistical analyses of data were performed using the Spss 20.0 software. The data were analyzed utilizing a paired *t*-test, with *p* values of <0.05 being considered statistically significant, and the data are represented as mean ± SD.

## 3. Results and Discussion

### 3.1. Optimization of Coaxial Electrospinning Process

Due to the concrete relationship between the electrospinning parameters and the resultant nanofiber properties [38,39], in particular, the diameters of nanofibers have some decisive influence on drug release profiles [40], all electrospinning parameters were carefully studied for preparation of emodin-loaded nanofiber membrane with core-shell structure. Considering the evaporation rate of electrospinning solutions is an important factor for controlling the morphology and electrospinnability of nanofibers, and emodin is highly hydrophobic, we tried several different solvents to prepare core solution and shell solution, such as anhydrous alcohol, acetone, mixture of acetone and DMAC (1:1, *v*/*v*), and mixture of acetone and DMAC (2:1, *v*/*v*) to dissolve CA, anhydrous alcohol, 0.2% dimethyl sulfoxide in anhydrous alcohol, and mixture of acetone and DMAC (2:1, *v*/*v*) to dissolve PVP with emodin; however, only the mixture of acetone and DMAC (2:1, *v*/*v*) showed optimal effect based on the electrospinnability, the morphology, and diameter distribution of the nanofibers containing emodin, thus the mixture of acetone and DMAC (2:1, *v*/*v*) was selected as the solvent to prepare the electrospinning solutions. Additionally, this solvent mixture showed acceptable biocompatibility on the basis of the preliminary cell viability evaluation. To ensure the electrospinnability of the solutions and to gain a biphasic release profile of emodin from the nanofibers, systematic investigations were carried out to optimize the PVP and CA concentration as well as the flow rate of core solution and shell solution. The optimum conditions were concluded: core solution, 13% PVP containing 8.5% emodin dissolved in the solvent mixture of acetone and DMAC (2/1, *v*/*v*); shell solution, 3% CA dissolved in the solvent mixture of acetone and DMAC (2/1, *v*/*v*); voltage, 11.0–12.5 kV; flow rate of solutions, 0.3 mm/min in 2.5 mL syringes with a coaxial metallic needle of 14G; and receiving distance, 15 cm. All the nanofibers discussed herein were prepared using the aforementioned optimum conditions.

### 3.2. Morphology and Core-Shell Structure of Electrospun Nanofibers

**Figure 2:**
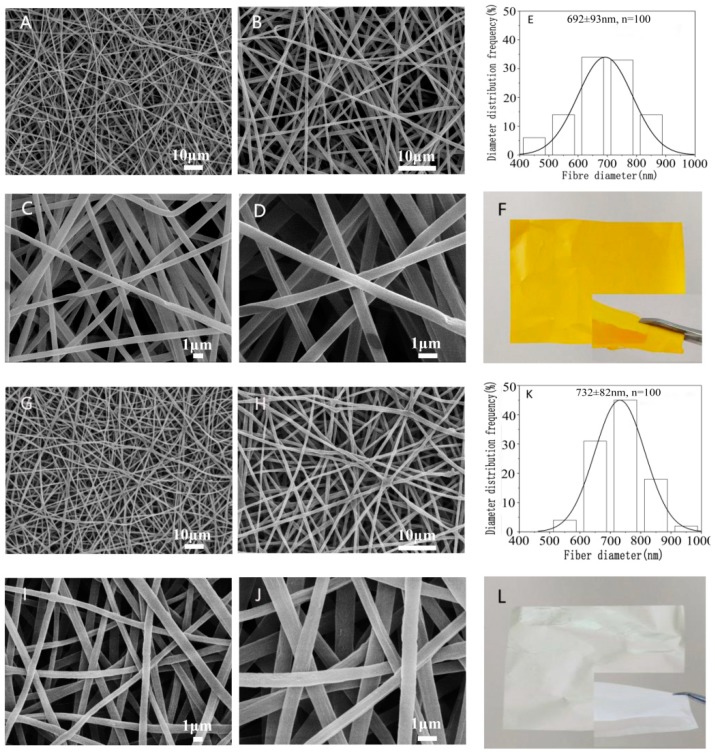
Morphology and diameter distribution of nanofiber membranes with/without emodin. Representative SEM images of emodin-loaded nanofibers (**A**–**D** observed at 1000×, 2000×, 5000×, and 10,000×, respectively); control nanofibers without emodin (**G**–**J** observed at 1000×, 2000×, 5000×, and 10,000×, respectively); diameter distribution histogram of emodin-loaded nanofibers (**E**) and control nanofibers without emodin (**K**); photographs of emodin-loaded nanofibers (**F**) and control nanofibers without emodin (**L**).

The morphology and diameter distribution of the nanofibers with and without emodin were summarized in Figure 2. The results of SEM showed that the nanofibers were homogeneous without beads and droplets, and the surface of the fibers was relatively smooth, indicating that the electrospinning conditions are reasonably good. The average diameters of nanofibers with and without emodin were 692 ± 93 nm and 732 ± 82 nm, respectively. While the diameters were significantly different (*p* < 0.05) by comparative analysis; the decreased diameters of nanofibers containing emodin are likely due to the increased conductivity of electrospinning solution with emodin, whose structure has three hydroxyls and two carbonyl groups possibly contributing to this effect, because the higher electrical conductivity leads to higher starching forces in the jet fluid, thus decreasing fiber diameters [39].

**Figure 3:**
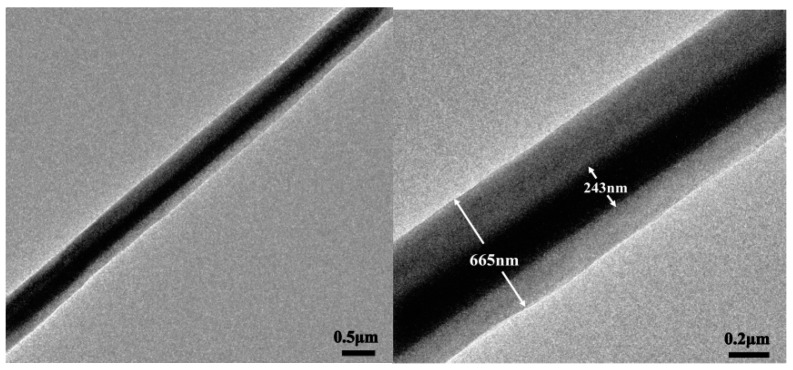
TEM observation of core-shell structure nanofiber containing emodin.

The core-shell structure of nanofibers containing emodin was further confirmed by TEM observations as shown in Figure 3. The diameter of the core layer was 223 ± 31 nm, which was about one third of the whole diameter, probably due to the same flow rates of core solution and shell solution.

### 3.3. Structural Characterization of Electrospun Nanofibers

#### 3.3.1. X-ray Diffraction

**Figure 4:**
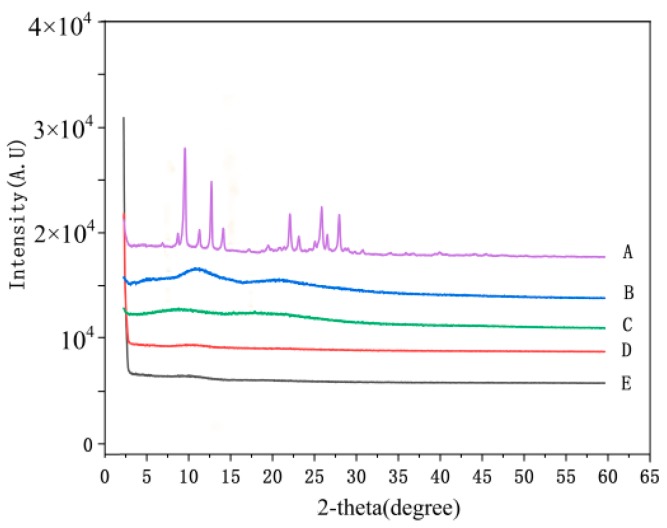
XRD patterns of nanofibers and raw materials. A: emodin; B: PVP; C: CA; D: nanofibers without emodin; E: nanofibers with emodin.

The dispersion state of drugs in the matrix is very key for nanofibers containing drugs, and the molecular-level dispersion of drug is an essential characteristic for drug delivery systems. In this study, emodin is a crystalline drug; in the XRD analysis, the results showed there were obvious diffraction peaks of emodin (shown in Figure 4), which confirmed the crystalline form of emodin raw material, whereas PVP and CA showed an amorphous pattern with two broad envelops. Interestingly, no obvious diffraction peaks of nanofibers with emodin, indicating that emodin existed in amorphous form in the electrospun nanofibers, which also indicated the molecular-level dispersion of emodin in the nanofibers. The possible reason is that the strong intermolecular interactions between emodin and PVP destroy the crystalline structure of emodin, resulting in the amorphous structure in the nanofibers.

#### 3.3.2. Differential Scanning Calorimetry (DSC)

**Figure 5:**
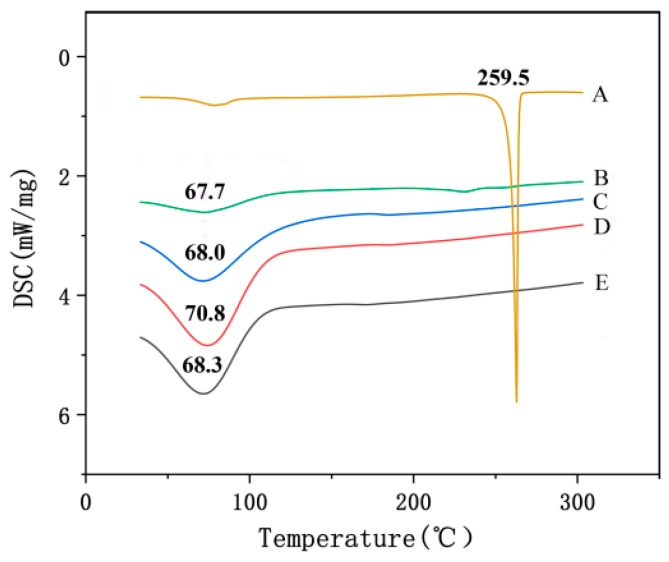
DSC thermograms of nanofibers and raw materials. A: emodin; B: CA; C: PVP; D: nanofibers without emodin; E: nanofibers with emodin.

As shown in Figure 5, the DSC thermogram of raw emodin showed a sharp endothermy peak at 259.5 °C, while the DSC curve of emodin-loaded nanofibers showed no endotherm peak of emodin, indicating that the crystalline of raw emodin was no longer present in the fibers but rather was converted into an amorphous state, which further confirmed the observation and conclusion from XRD data.

#### 3.3.3. ATR-FTIR Spectra Analysis

**Figure 6:**
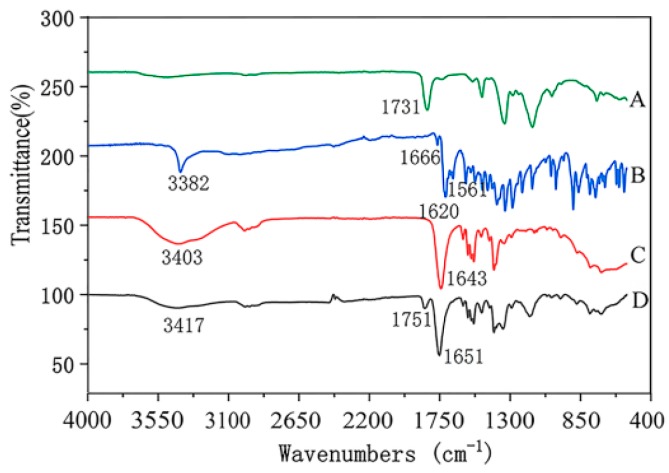
ATR-FTIR spectra of nanofibers and raw materials. A: CA; B: emodin; C: PVP; D: nanofibers with emodin.

FTIR spectra of nanofibers with emodin and raw materials were shown in Figure 6. The results showed that FTIR spectrum of raw emodin had two characteristic sharp peaks at 1620 cm^−1^ and 3382 cm^−1^, which is consistent with the standard infrared spectra of this compound, indicating the existence of emodin crystalline [26], while the characteristic peak at 1620 cm^−1^ of nanofibers containing emodin disappeared, additionally, most of the peaks in the fingerprint area of emodin-loaded fibers were different from those of raw emodin. FIIR results indicated that the hydrogen bond between PVP and emodin was formed, and emodin was distributed in nanofibers at a molecular level instead of crystalline lattice, which also indicated the stability of dispersions in electrospinning matrix. The results of FTIR also were consistent with those of XRD and DSC analysis, which also confirmed the high dispersibility of the electrospun nanofibers containing emodin.

### 3.4. Entrapment Efficiency

The standard curve of emodin was y = 0.0703x − 0.011 (r = 0.9999), which indicated that emodin had a good liner relationship within the concentration range 1.96–17.60 μg/mL. The average entrapment efficiency of the nanofiber membranes containing emodin was 99.38 ± 1.00%, which indicated that the electrospinning nanofibers had a high entrapment efficiency, and the specific results were shown in Table 1.

### 3.5. In Vitro Release Profiles

**Figure 7:**
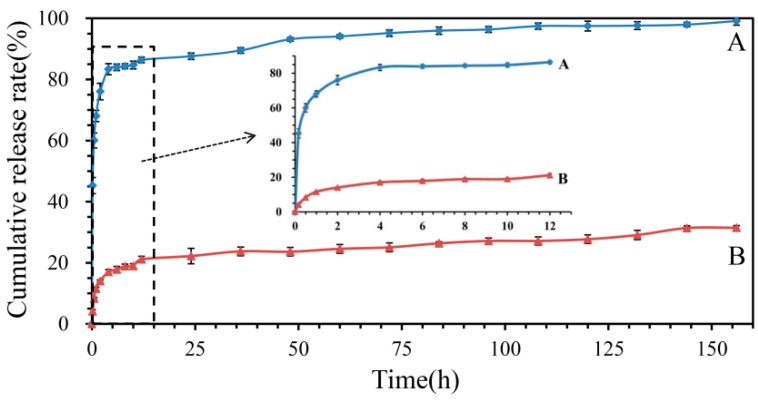
Cumulative release curves of emodin from nanofibers and raw emodin. A: nanofibers containing emodin, B: raw emodin. The insert profiles show the release behavior of nanofibers and raw emodin during the initial 12 h.

The release profiles of emodin in nanofiber membranes and raw emodin were shown in Figure 7. It showed that the dissolution rate of emodin from the nanofiber membranes was significantly higher than that of raw emodin (*p* < 0.001), for example, within 1, 12, and 156 h, 62.02 ± 2.18%, 85.55 ± 0.67% and 99.43 ± 0.32% of emodin from the membranes was released, respectively, while only 11.53 ± 0.51%, 24.14 ± 1.01%, and 31.47 ± 0.70% raw emodin was dissolved in the same period. Obviously, the release curve of the emodin-loaded nanofibers exhibited an abrupt release within 0.5 h (49.15 ± 2.89%) and then shifted to a steady release for the next 6 days achieving about 100% release, which indicated that the drug initially underwent rapid release then turned into a period of sustained release. This kind of release profile is essential in some cases of disease treatment, such as to reduce pain rapidly upon administration of the drug, and then to prolong analgesia through sustained release in order to provide a curative effect over the long term [41]. The biphasic release profile of emodin-loaded nanofiber membranes could be described as a quick release in the initial stage as a result of the hydrophilicity of PVP matrix, while the shell layer of CA matrix delays the emodin release in the drug delivery system, which is very different from the blending emodin-loaded PVP electrospinning delivery system [25]. While the low water solubility of raw emodin caused the low release degree in PBS solution.

### 3.6. Swelling Ratio

**Figure 8:**
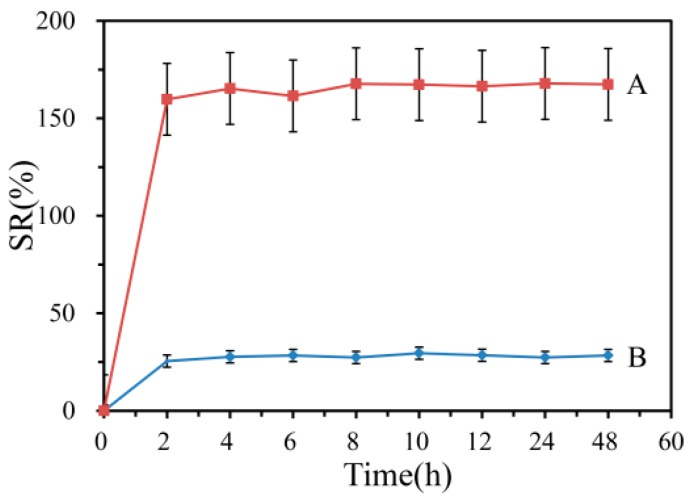
Swelling ratio of electrospinning nanofibers. A: nanofibers with emodin; B: nanofibers without emodin.

The swelling ratio of nanofiber membranes with and without emodin were shown in Figure 8. The results showed that the nanofiber membranes without emodin had the maximum swelling ratio of 29.5%, while the emodin-loaded nanofiber membranes showed swelling ratio of 167.8%, which indicated the presence of emodin showed a noticeable effect on the membrane swelling. This significant increment in SW% (*p* < 0.001) could be attributed to the increased hydrogen bonding between the water molecules and emodin [42]. The good swelling property of emodin-loaded nanofibers could maintain a better wound-healing environment.

### 3.7. Cell Proliferation Assay

**Figure 9:**
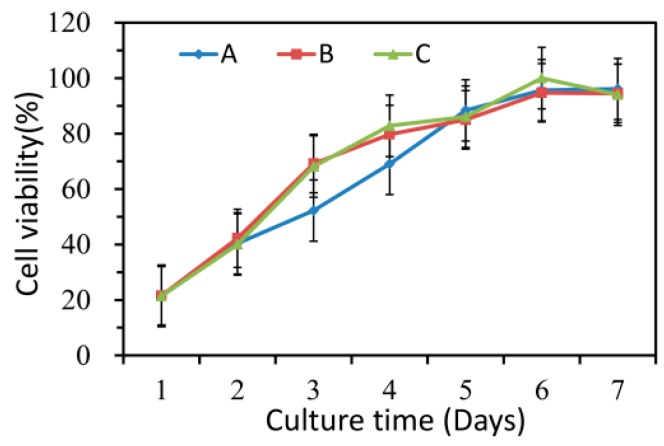
Cell compatibility of electrospinning nanofibers. A: control; B: nanofiber membranes without emodin; C: nanofiber membranes with emodin.

**Figure 10:**
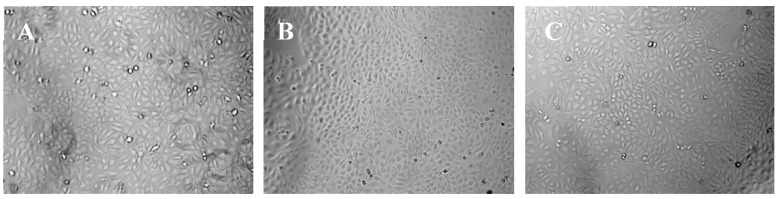
Representative microscopy images of HaCaT cells after 3 days incubation. (**A**) control; (**B**) nanofiber membranes without emodin; (**C**) nanofiber membranes with emodin.

The results of CCK-8 assays were shown in Figure 9, which indicated that the HaCaT cells treated with nanofiber membranes with and without emodin both exhibited normal growth compared with those of the control samples (*p* > 0.05). These results also indicated that the nanofibers had no toxicity on HaCaT cells during the incubation period. Figure 10 showed the microscopy images of the control, nanofibers with and without emodin after 72 h incubation. It was to observe the morphology of the HaCaT cells, in which most of the cells were normal. Thus, the cell compatibility of the fabricated coaxial electrospinning nanofibers is acceptable.

### 3.8. Antimicrobial Activity

**Figure 11:**
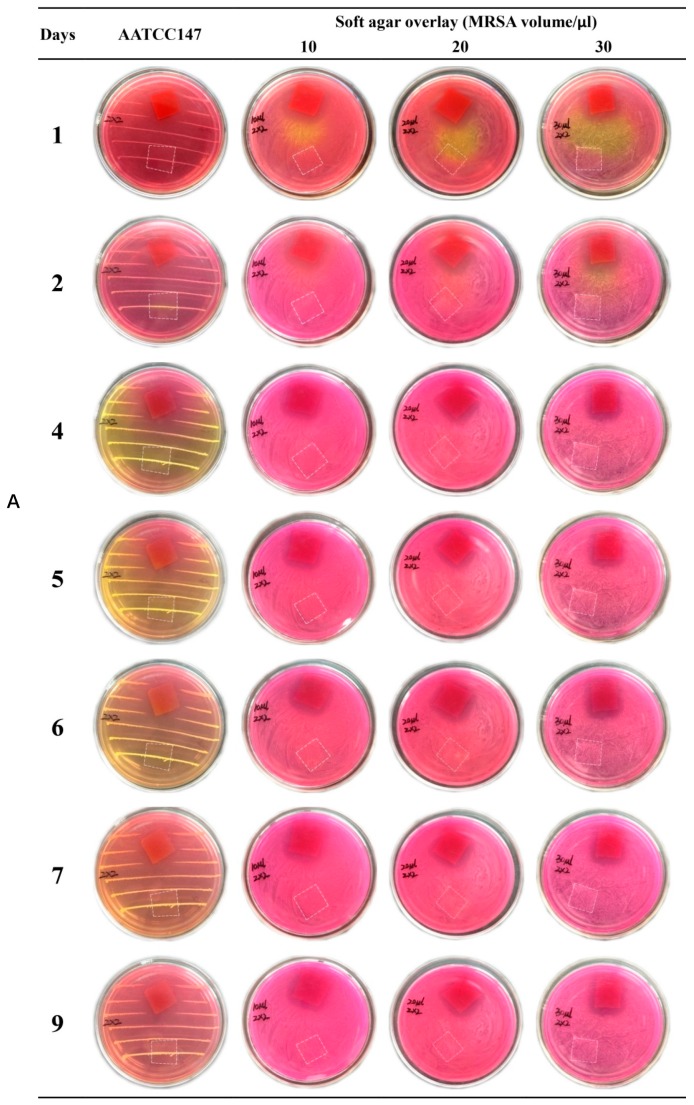

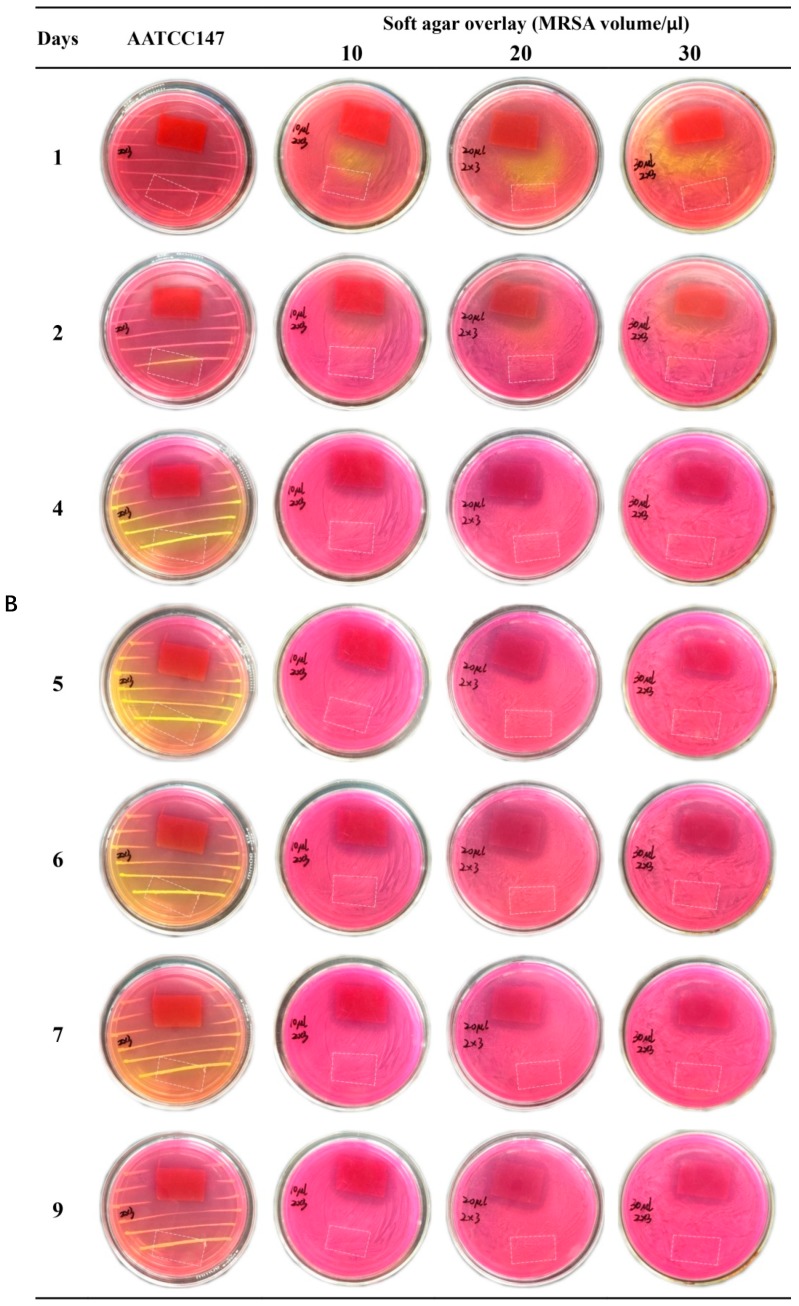
Evaluation of anti-MRSA of nanofiber membranes. Red membranes ((**A**) 2 × 2 cm; (**B**) 2 × 3 cm) in the petri dishes: nanofibers with emodin; area marked by white dotted box ((**A**) 2 × 2 cm; (**B**) 2 × 3 cm) in the petri dishes: nanofibers without emodin.

Emodin is an anthraquinone compound containing three hydroxyl groups, which shows color changes at different pH from yellow in acidic conditions to red in basic conditions [43]. Therefore, the nanofiber membranes with emodin became red after overlaying on the MSA (pH 7.4) (shown in Figure 11), while nanofiber membranes without emodin still kept white color (marked by white dotted box in Figure 11 to indicate the boundary). MSA is selected as the culture medium because it is a selective differential medium for *S. aureus*; the acid produced by *S. aureus* at enough density growing on MSA plate would react with the phenol red dye in the medium and change color from red to yellow [35]. On the plate prepared by AATCC test method 147, peripheral zones of growing colonies along the streaking lines were changed to yellow color, while the areas without growing biomass kept red (Figure 11). On the soft agar overlay, although the bacterial lawn spread all over the plates, most areas of the overlay were still red at three different adding amounts of MRSA ATCC 43300, probably because of the low density of biomass of MRSA. Obviously, MRSA ATCC 43300 grew intact under the nanofiber membranes without emodin in both methods. In contrast, clear inhibition zones around the nanofiber membranes with emodin were observed after incubated for 24 h, and up to 9 days without size reduction of inhibition zones, which was possibly related to the biphasic release profile with an initial rapid release to reach bacteriostatic concentration, followed by a slower sustained release for up to 156 h to keep bacteriostatic concentration. The results indicated the coaxial electrospinning nanofibers containing emodin have promising long-term effect against MRSA.

## 4. Conclusions

In this study, the bead-free and uniform nanofibers containing emodin in core shell were obtained using coaxial electrospinning mode, and the analysis results of XRD, DSC, and ATR-FTIR showed clearly the amorphous state of emodin in the nanofibers. The diameters in nanometer size and the amorphous state of emodin in the fibers significantly improved the release rate of emodin in nanofibers in PBS solution (pH 7.4), especially compared with the raw emodin. CCK-8 assay indicated that the emodin-loaded nanofiber membrane showed good compatibility on the HaCaT cells. Interestingly, the long-term effect against MRSA ATCC 43300 of the emodin-loaded nanofibers with biphasic release profile could be significantly proved by the clear inhibition zones from 24 h up to 9 days. Additionally, the nanofiber membranes retained integrity during the extended antimicrobial activity experiments and in vitro release evaluation because of the excellent film-forming property of CA. In conclusion, all the results indicate the developed nanofiber membranes containing emodin have potential as wound dressings with long-term effect against methicillin-resistant *Staphylococcus aureus*.

## Figures and Tables

**Table 1:** Entrapment efficiency of emodin in nanofiber membrane (n = 4).

No	Membrane Weight/mg	Theoretical Quantity of Emodin/μg	Determined Quantity of Emodin/μg	Entrapment Efficiency/%	Mean ± SD %
**1**	5.48	352.87	345.20	97.82	
**2**	4.95	318.75	315.26	98.91	99.38 ± 1.00
**3**	5.30	342.39	332.76	97.19	
**4**	5.10	349.47	327.44	99.38	

## References

[1] Lee A.S., De Lencastre H., Garau J., Kluytmans J., Malhotra-Kumar S., Peschel A., Harbarth S. (2018). Methicillin-resistant Staphylococcus aureus. Nat. Rev. Dis. Prim..

[2] Hayden M.K., Rezai K., Hayes R.A., Lolans K., Quinn J.P., Weinstein R.A. (2005). Development of Daptomycin Resistance In Vivo in Methicillin-Resistant Staphylococcus aureus. J. Clin. Microbiol..

[3] Talan D.A., Krishnadasan A., Gorwitz R.J., Fosheim G.E., Limbago B., Albrecht V., Moran G.J. (2011). Comparison of Staphylococcus aureus From Skin and Soft-Tissue Infections in US Emergency Department Patients, 2004 and 2008. Clin. Infect. Dis..

[4] Norbury W., Herndon D.N., Tanksley J., Jeschke M.G., Finnerty C.C. (2016). Infection in Burns. Surg. Infect..

[5] Church D.L., Elsayed S., Reid O., Winston B., Lindsay R. (2006). Burn Wound Infections. Clin. Microbiol. Rev..

[6] Tejiram S., Johnson L.S., Mete M., Desale S., Johnson K., Zhang J., Moffatt L.T., Shupp J.W. (2017). Screening nasal swabs for methicillin resistant Staphylococcus aureus: A regional burn center’s experience. Burns.

[7] Loomba P.S., Taneja J., Mishra B. (2010). Methicillin and Vancomycin Resistant S. aureus in Hospitalized Patients. J. Glob. Infect. Dis..

[8] Gupta S.C., Rai V. (2018). Role of Emodin in Chemosensitization of Cancer.

[9] Zin W.W.M., Buttachon S., Dethoup T., Pereira J., Gales L., Inácio Â., Da Costa P.M., Lee M., Sekeroglu N., Silva A.M.S. (2017). Antibacterial and antibiofilm activities of the metabolites isolated from the culture of the mangrove-derived endophytic fungus Eurotium chevalieri KUFA 0006. Phytochemistry.

[10] Ji X., Liu X., Peng Y., Zhan R., Xu H., Ge S. (2017). Comparative analysis of methicillin-sensitive and resistant Staphylococcus aureus exposed to emodin based on proteomic profiling. Biochem. Biophys. Res. Commun..

[11] Haque E., Kamil M., Irfan S., Sheikh S., Hasan A., Nazir A., Mir S.S. (2018). Blocking mutation independent p53 aggregation by emodin modulates autophagic cell death pathway in lung cancer. Int. J. Biochem. Cell Biol..

[12] Shia C.-S., Hou Y.-C., Tsai S.-Y., Huieh P.-H., Leu Y.-L., Chao P.-D.L. (2010). Differences in pharmacokinetics and ex vivo antioxidant activity following intravenous and oral administrations of emodin to rats**Chi-Sheng Shia and Yu-Chi Hou contributed equally to this work. J. Pharm. Sci..

[13] Park S.Y., Jin M.L., Ko M.J., Park G., Choi Y.-W. (2016). Anti-neuroinflammatory Effect of Emodin in LPS-Stimulated Microglia: Involvement of AMPK/Nrf2 Activation. Neurochem. Res..

[14] Han J.-W., Shim D.-W., Shin W.-Y., Heo K.-H., Kwak S.-B., Sim E.-J., Jeong J.-H., Kang T.-B., Lee K.-H. (2015). Anti-Inflammatory Effect ofEmodin via Attenuation of NLRP3 Inflammasome Activation. Int. J. Mol. Sci..

[15] Davidov-Pardo G., McClements D.J. (2014). Resveratrol encapsulation: Designing delivery systems to overcome solubility, stability and bioavailability issues. Trends Food Sci. Technol..

[16] Liu W., Tang L., Ye L., Cai Z., Xia B., Zhang J., Hu M., Liu Z. (2010). Species and Gender Differences Affect the Metabolism of Emodin via Glucuronidation. AAPS J..

[17] Wang B., Chen L., Sun Y., Zhu Y., Sun Z., An T., Li Y., Lin Y., Fan D., Wang Q. (2015). Development of phenylboronic acid-functionalized nanoparticles for emodin delivery. J. Mater. Chem. B.

[18] Ban E., Park M., Jeong S., Kwon T., Kim E.-H., Jung K., Kim A. (2017). Poloxamer-Based Thermoreversible Gel for Topical Delivery of Emodin: Influence of P407 and P188 on Solubility of Emodin and Its Application in Cellular Activity Screening. Molecules.

[19] Shi Y., Li H., Li J., Zhi D., Zhang X., Liu H., Wang H., Li H. (2015). Development, optimization and evaluation of emodin loaded nanoemulsion prepared by ultrasonic emulsification. J. Drug Deliv. Sci. Technol..

[20] Chen X., Yang Z., Sun R., Mo Z., Jin G., Wei F., Hu J., Guan W., Zhong N.-S. (2014). Preparation of Lung-Targeting, Emodin-Loaded Polylactic Acid Microspheres and Their Properties. Int. J. Mol. Sci..

[21] Jangra S., Chhokar V., Tomer V.K., Sharma A.K., Duhan S. (2016). Influence of functionalization type on controlled release of emodin from mesoporous silica. J. Porous Mater..

[22] Xu Y., Wang C., Zhou G., Wu Y., Chen J. (2012). Improving the controlled release of water-insoluble emodin from amino-functionalized mesoporous silica. Appl. Surf. Sci..

[23] Tao J.J., Xu Y.Q., Zhou G.W., Wu C.C., Bin Song H., Wang C.F. (2011). Ordered Mesoporous SBA-15 for Controlled Release of Water-Insolube Drug. Adv. Mater. Res..

[24] Campos P.P., Fraceto L., Ferreira M. (2018). Layer-by-layer films containing emodin or emodin encapsulated in liposomes for transdermal applications. Colloids Surf. B: Biointerfaces.

[25] Dai X.-Y., Nie W., Wang Y.-C., Shen Y., Li Y., Gan S.-J. (2012). Electrospun emodin polyvinylpyrrolidone blended nanofibrous membrane: A novel medicated biomaterial for drug delivery and accelerated wound healing. J. Mater. Sci. Mater. Electron..

[26] Greiner A., Wendorff J.H. (2007). Electrospinning: A fascinating method for the preparation of ultrathin fibers. Angew. Chem. Int. Ed. Engl..

[27] Sill T.J., Von Recum H.A. (2008). Electrospinning: Applications in drug delivery and tissue engineering. Biomaterials.

[28] Sperling L.E., Reis K.P., Pranke P., Wendorff J. (2016). Advantages and challenges offered by biofunctional core–shell fiber systems for tissue engineering and drug delivery. Drug Discov. Today.

[29] Calamak S., Shahbazi R., Eroglu I., Gultekinoglu M., Ulubayram K. (2017). An overview of nanofiber-based antibacterial drug design. Expert Opin. Drug Discov..

[30] Wang J., Windbergs M. (2019). Controlled and dual drug release by conaxial electrospun fibers-Impact of the core fluid on drug encapsulation and release. Int. J. Pharm..

[31] Sriyanti I., Edikresnha D., Rahma A., Munir M.M., Rachmawati H., Khairurrijal K. (2018). Mangosteen pericarp extract embedded in electrospun PVP nanofiber mats: Physicochemical properties and release mechanism of α-mangostin. Int. J. Nanomed..

[32] Godakanda V.U., Li H., Alquezar L., Zhao L., Zhu L.-M., De Silva R., De Silva K.M.N., Williams G.R. (2019). Tunable drug release from blend poly(vinyl pyrrolidone)-ethyl cellulose nanofibers. Int. J. Pharm..

[33] Khoshnevisan K., Maleki H., Samadian H., Shahsavari S., Sarrafzadeh M.H., Larijani B., Dorkoosh F.A., Haghpanah V., Khorramizadeh M.R. (2018). Cellulose acetate electrospun nanofibers for drug delivery systems: Applications and recent advances. Carbohydr. Polym..

[34] Yang Y., Li W., Yu D.-G., Wang G., Williams G.R., Zhang Z. (2019). Tunable drug release from nanofibers coated with blank cellulose acetate layers fabricated using tri-axial electrospinning. Carbohydr. Polym..

[35] Han D., Sherman S., Filocamo S., Steckl A.J. (2017). Long-term antimicrobial effect of nisin released from electrospun triaxial fiber membranes. Acta Biomater..

[36] Li X., Wang C., Yang S., Liu P., Zhang B. (2018). Electrospun PCL/mupirocin and chitosan/lidocaine hydrochloride multifunctional double layer nanofibrous scaffolds for wound dressing applications. Int. J. Nanomed..

[37] Yun J., Im J.S., Lee Y.S., Kim H.-I. (2011). Electro-responsive transdermal drug delivery behavior of PVA/PAA/MWCNT nanofibers. Eur. Polym. J..

[38] Zhou H., Shi Z., Wan X., Fang H., Yu D.-G., Chen X., Liu P. (2019). The Relationships between Process Parameters and Polymeric Nanofibers Fabricated Using a Modified Coaxial Electrospinning. Nanomaterials.

[39] Wang M., Hai T., Feng Z., Yu D.-G., Yang Y., Annie Bligh S.W. (2019). The Relationships between the Working Fluids, Process Characteristics and Products from the Modified Coaxial Electrospinning of Zein. Polymers.

[40] Yang Y., Zhu T., Liu Z.-P., Luo M., Yu D.-G., Bligh S.A. (2019). The key role of straight fluid jet in predicting the drug dissolution from electrospun nanofibers. Int. J. Pharm..

[41] Murakami H., Kobayashi M., Takeuchi H., Kawashima Y. (2000). Utilization of poly(dllactide-co-Glycolide) nanoparticles for preparation of mini-depot tablets by direct compression. J. Control. Release.

[42] Abid S., Hussain T., Nazir A., Zahir A., Ramakrishna S., Hameed M., Khenoussi N. (2019). Enhanced antibacterial activity of PEO-chitosan nanofibers with potential application in burn infection management. Int. J. Biol. Macromol..

[43] Duval J., Pecher V., Poujol M., Lesellier E. (2016). Research advances for the extraction, analysis and uses of anthraquinones: A review. Ind. Crops Prod..

